# Experimental Challenge of Atlantic Cod (*Gadus morhua*) with a *Brucella pinnipedialis* Strain from Hooded Seal (*Cystophora cristata*)

**DOI:** 10.1371/journal.pone.0159272

**Published:** 2016-07-14

**Authors:** Ingebjørg Helena Nymo, Marit Seppola, Sascha Al Dahouk, Kathrine Ryvold Bakkemo, María Pilar Jiménez de Bagüés, Jacques Godfroid, Anett Kristin Larsen

**Affiliations:** 1 Arctic Infection Biology, Department of Arctic and Marine Biology, UiT–The Arctic University of Norway, Tromsø, Norway; 2 Department of Medical Biology, UiT–The Arctic University of Norway, Tromsø, Norway; 3 Federal Institute for Risk Assessment, Berlin, Germany; 4 RWTH Aachen University, Department of Internal Medicine III, Aachen, Germany; 5 PHARMAQ AS, Oslo, Norway; 6 Unidad de Tecnología en Producción y Sanidad Animal, Centro de Investigación y Tecnología Agroalimentaria (CITA), Instituto Agroalimentario de Aragón–IA2 (CITA–Universidad de Zaragoza), Zaragoza, Spain; Purdue University, UNITED STATES

## Abstract

Pathology has not been observed in true seals infected with *Brucella pinnipedialis*. A lack of intracellular survival and multiplication of *B*. *pinnipedialis* in hooded seal (*Cystophora cristata*) macrophages *in vitro* indicates a lack of chronic infection in hooded seals. Both epidemiology and bacteriological patterns in the hooded seal point to a transient infection of environmental origin, possibly through the food chain. To analyse the potential role of fish in the transmission of *B*. *pinnipedialis*, Atlantic cod (*Gadus morhua*) were injected intraperitoneally with 7.5 x 10^7^ bacteria of a hooded seal field isolate. Samples of blood, liver, spleen, muscle, heart, head kidney, female gonads and feces were collected on days 1, 7, 14 and 28 post infection to assess the bacterial load, and to determine the expression of immune genes and the specific antibody response. Challenged fish showed an extended period of bacteremia through day 14 and viable bacteria were observed in all organs sampled, except muscle, until day 28. Neither gross lesions nor mortality were recorded. Anti-*Brucella* antibodies were detected from day 14 onwards and the expression of hepcidin, cathelicidin, interleukin (IL)-1β, IL-10, and interferon (IFN)-γ genes were significantly increased in spleen at day 1 and 28. Primary mononuclear cells isolated from head kidneys of Atlantic cod were exposed to *B*. *pinnipedialis* reference (NCTC 12890) and hooded seal (17a-1) strain. Both bacterial strains invaded mononuclear cells and survived intracellularly without any major reduction in bacterial counts for at least 48 hours. Our study shows that the *B*. *pinnipedialis* strain isolated from hooded seal survives in Atlantic cod, and suggests that Atlantic cod could play a role in the transmission of *B*. *pinnipedialis* to hooded seals in the wild.

## Introduction

*Brucella* spp. were isolated from marine mammals in 1994 [1] and published as *Brucella pinnipedialis* and *Brucella ceti* in 2007 [2]. Marine mammal brucellae have been isolated from several pinniped and cetacean species, and although *B*. *ceti*-associated pathology is well documented in dolphins, reported pathology associated with infection of true seals with *B*. *pinnipedialis* is sparse [3]. Hooded seals (*Cystophora cristata*) of the Northeast Atlantic stock have a high prevalence of *Brucella* (serology and bacteriology), but pathological changes due to infection with *B*. *pinnipedialis* hooded seal (HS) strain have not been observed [4, 5].

Pathology may occur in other mammals following experimental infection with *B*. *pinnipedialis*, although reports are limited. No pathology was observed in piglets (*Sus scrofa domesticus*) [6, 7], limited pathology was detected in pregnant sheep (*Ovis aries*) [8], but infection of guinea pigs (*Cavia porcellus*) resulted in splenomegaly and high antibody levels [8]. In the BALB/c mouse (*Mus musculus*) model of infection, a *B*. *pinnipedialis* HS strain had lower pathogenicity than *Brucella suis* 1330 [9], and the *B*. *pinnipedialis* reference strain was found to be attenuated [10]. The only severe pathological outcome has been identified in cattle (*Bos taurus*), where abortion was induced after infection with a *B*. *pinnipedialis* Pacific harbour seal (*Phoca vitulina richardsi*) strain [11].

Previous *in vitro* work has shown that *B*. *pinnipedialis* reference strain and *B*. *pinnipedialis* HS strain were eliminated from murine and human macrophage cell lines, and a human epithelial cell line within 72–96 h [12], and they were eliminated more rapidly from hooded seal primary alveolar macrophages [13]. The absence of survival in mononuclear phagocytic cells suggests that *B*. *pinnipedialis* might not be able to cause a chronic infection in seals. Additionally, the *B*. *pinnipedialis* HS strain was quickly eliminated from infected hooded seal peripheral blood mononuclear cells (PBMCs) (Larsen, unpublished data), as well as primary epithelial cells [14]. This absence of intracellular multiplication in primary hooded seal cells has raised doubts as to whether the hooded seal should be considered the primary host for *B*. *pinnipedialis* HS strain. Infection could be transmitted from hitherto unknown marine hosts rather than within the hooded seal population. *Brucella melitensis* has been isolated from Nile catfish (*Clarias gariepinus*) under natural conditions [15], while seroconversion and recovery of *B*. *melitensis* from visceral organs was shown in catfish after experimental infection [16]. The ecological range of brucellae has recently been extended to include ectotherms and the environment, with isolation of novel brucellae from frogs (*Ranidae*) [17–19] and *Brucella microti* from soil [20].

The lack of concurrent pathology in *Brucella*-positive true seals has puzzled wildlife scientists, and although a transmission route similar to terrestrial brucellosis is nearby to suspect, the infection pathway of *B*. *pinnipedialis* is unknown. There is no evidence for a chronic disease with vertical transmission. Age-dependent serological and bacteriological patterns for *B*. *pinnipedialis* have been identified in hooded seals. Pups have a low probability of being positive, whereas the probability for yearlings being positive is high, followed by a decreasing probability with age. This suggests post-weaning exposure during the first year of life followed by clearance of infection in older animals [4]. Similarly, age-dependent patterns of anti-*Brucella* antibodies have been found in harbour seals [21, 22]. Consequently, an environmental source of infection may be suspected with the possibility of a reservoir of *B*. *pinnipedialis* in the prey consumed by the seals. The diet of hooded seals consists of Atlantic (*Gadus morhua*) and polar cod (*Boreogadus saida*) along with a range of other species, such as deep-sea squid (*Gonatus fabricii*), redfish (*Sebastes* sp.), and Greenland halibut (*Reinhardtius hippoglossoides*) [23]. Fish have been identified as intermediate hosts for the most common species of lungworms in harbour seals [24] and *B*. *pinnipedialis* has been isolated from lungworms in pinnipeds [25], but to what extent lungworms play a role in transmission of *B*. *pinnipedialis* to pinnipeds is not known.

This study investigates the possible extended ecology of marine brucellae and aim to assess whether *B*. *pinnipedialis* HS strain may have gadid fish as a host. The infective capacity of *B*. *pinnipedialis* HS strain was studied in Atlantic cod by performing *in vitro* infection of head kidney derived macrophages and *in vivo* experimental infections.

## Materials and Methods

### Bacterial strains and growth conditions

The strains used were a *B*. *pinnipedialis* HS field isolate (strain 17a-1; [5]) and the *B*. *pinnipedialis* reference strain (NCTC 12890^T^, BCCN 94-73^T^) from harbour seal [2]. Bacteria were grown on Tryptic Soy Agar (TSA, Oxoid, Basingstoke, UK) at 37°C in an atmosphere of air plus 5% CO_2_, with the exception of fecal and water samples which were grown on modified Farrell medium (one vial of *Brucella* selective supplement (Oxoid) per TSA litre + 5% foetal calf serum (FCS)). The strains were kept at -80°C on Microbank™ beads (Pro-Lab Diagnostics, Round Rock, TX, USA). Before the infection a bead was plated and the bacteria were grown for 2–4 days and subsequently sub-cultured for 96 h.

### Atlantic cod head kidney derived monocytes/macrophages

Atlantic cod (approx. 150 g, *n* = 5, and approx. 1000 g, *n* = 4) were obtained from the Tromsø Aquaculture Research Station (TARS, Kårvika, Tromsø, Norway). Head kidney derived monocyte/macrophage-like cells (HKDM) [26] were isolated by density gradient sedimentation as described by [27].

### HKDM infection assay

Atlantic cod HKDM were seeded (approx. 10^7^ cells/well) in 24 well plates (Nunc PolySorp, Thermo Fisher Scientific Inc., Waltham, MA, USA) and prepared for the infection assay as described by [28], with some modifications. After 24 h, the medium was changed and the cells were washed twice with Leibovitz’s L-15 medium (Fisher Scientific) supplemented with 25 mM HEPES (Life Technologies, Carlsbad, CA, USA), 2 mM L-glutamine, 20.5 mM NaCl, 1.8 mM glucose, 4.2 mM NaHCO_3_, 20 U/ml penicillin and 20 mg/ml streptomycin (Sigma Aldrich, St. Louis, MO, USA) (L-15+) to remove non-adherent cells. The infection assay was initiated 48 h after initial seeding of the cells. Bacteria were diluted in L-15+, 5% FCS, without antibiotics to prepare the infective dose and the cells were infected with *Brucella* spp. at a multiplicity of infection (MOI) of 50 for 1 h and incubated at 10–12°C. The plates were centrifuged at 230 *x g* for 10 min at room temperature to facilitate contact between bacteria and the adherent HKDM cell monolayer. The infection was terminated by rinsing the wells twice with medium and refilling with 1 ml of L-15+, 5% FCS, containing 50 μg/ml gentamicin to kill extracellular bacteria. After 1 h the medium was replaced with L-15+, 5% FCS, containing 10 μg/ml gentamicin, in which the cells were incubated for specified periods of time. The infection of HKDM and harvesting of intracellular bacteria was performed as described by [13]. Potential toxic cell damage was measured by quantitatively determining the release of lactate dehydrogenase using the CytoTox 96 Non-Radioactive Cytotoxicity Assay (Promega, Madison, WI, USA) according to the manufacturer’s instructions. Absorbance was read using an Epoch Microplate Spectrophotometer (BioTek Instruments Inc., Winooski, VT, USA).

### Atlantic cod (*Gadus morhua*) for experimental challenge

Atlantic cod, which forms part of the diet of hooded seals, is easy to hold in captivity and is a farmed species for which optimal aquaculture conditions are well established. Consequently, cod was selected as the test species for this study. Atlantic cod (ca 150 g, *n* = 39) were purchased from Sagafjord Sea Farm AS and held at Tromsø Aquaculture Research Station in a 1000 L tank with filtered seawater for one week. During this week the temperature was increased from 4.5°C to 10°C. The fish were then divided into two groups and kept in two 500 L tanks under a 24L:0D photoperiod and feed commercial pellet (Amber Neptun Starter 5.0 mm, Skretting, Stavanger, Norway). Prior to infection and sampling (day 1, 7, 14 and 28) the fish were fasted for 48 hours and anesthetized with 0.08 g/L Metacain (Argent laboratories, WA, USA). The experiment was conducted in strict accordance with the Norwegian Animal Welfare Act and the regulations for use of animals in experimentation. The protocol was approved by the Norwegian Animal Research Authority (permit no. 6503). All efforts were made to minimize suffering and stress, both during handling and sampling, humane endpoints were used and any fish that showed signs of disease or abnormal behaviour (lethargy, bloating, disoriented swimming) was euthanized by a quick blow to the head followed by dislocation of the cervical vertebra. At all sampling times, fish were almost completely exsanguinated by blood sampling and euthanized using an overdose of Metacain before collection of organ samples.

### Experimental challenge

Dilutions of bacteria in sterile phosphate-buffered saline (sPBS) were used to prepare the infective doses. The expression of smooth surface antigens was verified by crystal violet staining and agglutination with antiserum to smooth *Brucella abortus* [29, 30]. The infected group (*n* = 21) received 7.5 x 10^7^ *B*. *pinnipedialis* HS field isolate 17a-1 in 100 μL sPBS intraperitoneally (ip). The control group (*n* = 18) received 100 μL sPBS ip.

### Sampling

Infected and control fish (*n* = 4–6) were sacrificed at day 1, 7, 14, and 28 post infection (pi). Blood was collected using vacutainer tubes without anticoagulant (BD Biosciences, San Jose, CA, USA), and allowed to clot over night at 4°C before centrifugation and collection of the sera. In addition, organ samples were taken sterilely for bacterial quantification including pieces of the spleen, liver, female gonads, heart, head kidney, and dorsal muscle. Feces from rectum were collected on sterile cotton swabs. Tissue and fecal samples were kept at -20°C until culture. Water samples were collected in vacutainer tubes without anticoagulant at all sampling times pi. The whole spleen and samples from different organs were weighed. Sub-samples from spleen were stored in RNAlater (Sigma-Aldrich, St. Louis, MO, USA) at -20°C.

### Bacterial quantification

All organ samples were manually homogenized, serially diluted in sPBS and plated on TSA to determine the number of colony forming units (CFU), while blood was plated (100 μl) directly. Fecal and water samples were plated on modified Farrell medium to detect possible bacterial shedding into the environment.

### Gene expression of cytokines and antibacterial peptides

Atlantic cod HKDM were seeded, infected and treated as described for the infection assay. The *in vitro* challenge was terminated by adding sample buffer (RNeasy Mini Kit, Qiagen, Venlo, Limburg, Netherlands) to the wells. RNA from HKDM and spleen was extracted (RNeasy Mini Kit) with on-column DNase digestion (RNase-Free DNase Set, Qiagen). An additional DNase digestion step was included after extraction to ensure absence of genomic DNA (RNeasy Mini Kit and RNase-Free DNase Set, Qiagen). The absence of genomic DNA was verified as described by [31]. RNA quality and quantity were assessed by measuring absorbance at 230, 260 and 280 nm (Nanodrop 2000, Thermo Fisher Scientific Inc.). An *A*_260_/*A*_280_ ratio ≥ 2.0 and an *A*_260_/*A*_230_ ratio ≥ 2.1 were considered acceptable. The cDNA was synthesized using 150 ng RNA (iScript™ cDNA Synthesis Kit, BioRad, Hercules, CA, USA). Primer sequences for hepcidin [32], cathelicidin [33], interleukin (IL)-1β, IL-10 [34], interferon (IFN)-γ [35], IL-12p40 [36] and ribosomal RNA (18S) [34] were retrieved from published sources. Real time PCR was performed in duplicates of 8 μl cDNA diluted 1/30, 10 μl iTaq Universal SYBR Green Supermix (BioRad), 0.6 μl of each primer (10 μM) and 0.8 μl DEPC water (Invitrogen) on a C1000 Thermal cycler, CFX96 Real-Time System (BioRad). Cycling parameters were set and threshold cycle (Ct) was calculated as described by [37]. Quantification of relative gene expression levels were performed using the 2 ^-ΔΔCT^ method [38]. Gene expression was calibrated against non-stimulated or non-injected controls from the same time pi, for HKDM and spleen, respectively, and data expressed as mean ± standard error of the mean (SEM).

### Enzyme-linked immunosorbent assay (ELISA)

The wells of 96-well polystyrene plates (Nunc PolySorp, Thermo Fisher Scientific Inc.) were coated with *B*. *abortus* lipopolysaccharide (LPS) as described by [39]. Cod sera (1:20, based on serial twofold dilutions of positive and negative sera 1:20–1:1280) were added to duplicate wells. Polyclonal rabbit anti-cod antibodies (1:800) were used as secondary antibodies [40], and goat anti-rabbit antibodies conjugated with horseradish peroxidase (1:2000) (Life Technologies) added thereafter [41]. Finally, o-phenylenediamine dihydrochloride (OPD, Sigma Aldrich) was diluted and hydrogen peroxide added, according to the recommendations of the producer. The reaction was terminated after 20 minutes in the dark, at room temperature, by adding 3 M sulfuric acid (H_2_SO_4_). The plates were washed between each step as described by [41]. Optical densities (OD) were measured at 492 (OD_492_) and 620 (OD_620_) nm using an Epoch Microplate Spectrophotometer (BioTek Instruments Inc.). The OD_620_ was subtracted from the OD_492_ for each well to normalize for disturbance from nonspecific components. A sample dilution buffer control, along with serum from a non-infected and an infected cod taken at day 28 pi were included on all plates.

### Statistics

Statistical analyses were performed with a paired, one-tailed (*in vitro* results) and an unpaired, one-tailed (*in vivo* results) Student *t*-test (*p* < 0.05 was considered significant).

## Results

### *Brucella pinnipedialis* survives in Atlantic cod head kidney derived monocytes/macrophages

**Fig 1:**
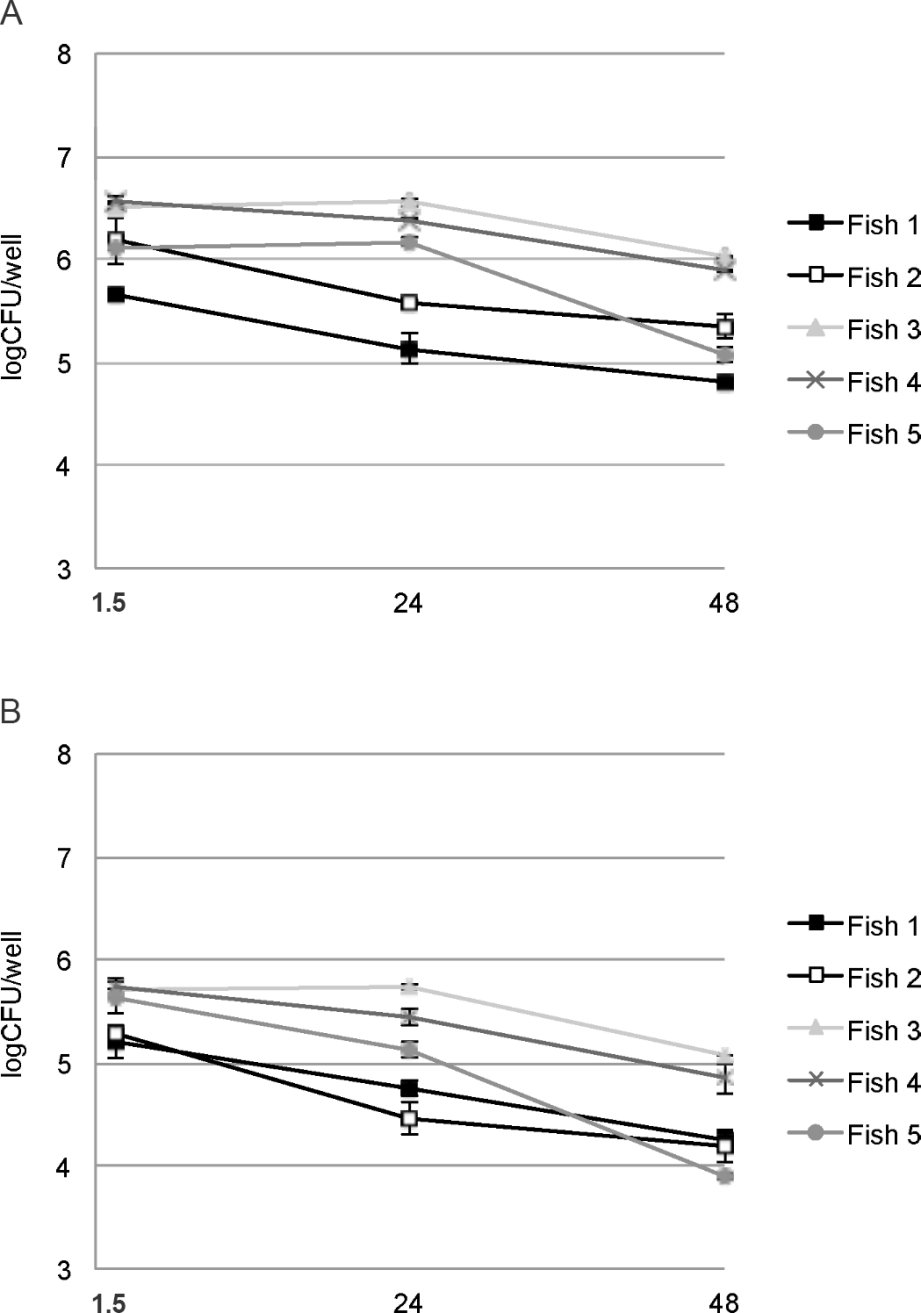
*Brucella pinnipedialis* survives in Atlantic cod head kidney derived monocytes/macrophages (HKDM). Intracellular survival of *B*. *pinnipedialis* hooded seal strain 17a-1 (A) and *B*. *pinnipedialis* reference strain 12890 (B) in HKDM at 1.5, 24, and 48 h post infection. Results from each fish are depicted individually and each time point represents the mean of 2 wells ± standard deviation.

Both *B*. *pinnipedialis* strains were able to enter Atlantic cod HKDM *in vitro* (Fig 1, S1 Table). Challenging HKDM harvested from small fish (150 g) with a MOI of 50 lead to recovery of 5.20 to 6.57 log CFU bacteria at 1.5 h pi. Elimination of intracellular *B*. *pinnipedialis* HS strain 17a-1 was slow and bacterial numbers at 48 h pi were 4.81 to 5.88 log CFU. Although recovery of intracellular *B*. *pinnipedialis* reference strain 12890 was significantly lower than 17a-1 at all times, there were no significant differences in the rates of elimination of the two strains. Although numbers of retrievable bacteria varied between individual fish, the pattern of intracellular entry and survival was similar for all fish investigated. Intracellular persistence of 17a-1 in HKDM harvested from larger fish (1000 g) was similar to that of smaller fish (S1 Fig). The release of lactate dehydrogenase from HKDM increased with time in culture and cellular integrity was impaired after 48 h (S2 Fig), but no significant differences were found between control cells, and cells infected with the reference (12890) or the hooded seal (17a-1) strain.

### *Brucella pinnipedialis* HS strain causes a disseminated infection in Atlantic cod

**Fig 2:**
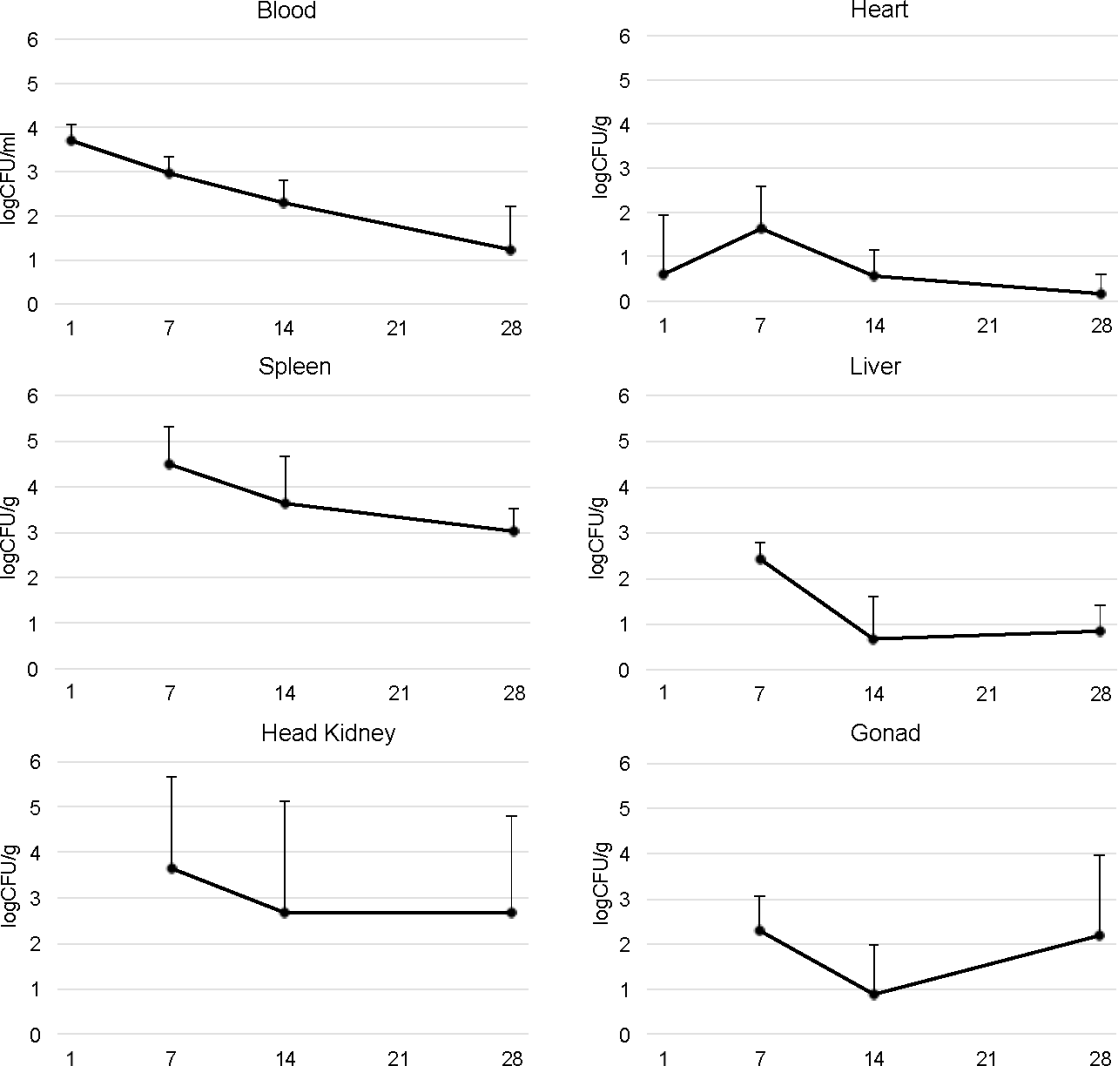
Atlantic cod infected with *Brucella pinnipedialis* show bacterial dissemination in multiple tissues. Number of colony forming units (CFU)/ml blood and CFU/g heart, spleen, liver, head kidney, and gonads from Atlantic cod after intraperitoneal injection of 7.5 x 10^7^ CFU of *B*. *pinnipedialis* hooded seal strain 17a-1. Infected fish were euthanized and sampled at day 1, 7, 14, and 28 post infection. Each indicator shows the mean log CFU ± standard deviation of *n* = 5 fish (day 1, 7, and 14) and *n* = 6 fish (day 28) for blood, spleen, liver, head kidneys, and heart. For gonads *n* = 5, 4 and 5 fish, for day 7, 14, and 28 pi, respectively.

*Brucella pinnipedialis* HS strain was found in all investigated tissues, except muscle (Fig 2, S1 Table)). On day 1 pi bacteria in blood and heart had a log CFU (mean ± SD) of 3.72 ± 0.36/ml and 0.60 ± 1.35/g, respectively. Infected Atlantic cod showed prolonged bacteremia and *B*. *pinnipedialis* HS strain was found in the blood at all times pi (2.99 ± 0.35, 2.30 ± 0.53, and 1.23 ± 0.98 log CFU/ml on days 7, 14, and 28 pi, respectively). On day 7 pi, bacteria present in tissues were: spleen 4.49 ± 0.81, head kidneys 3.63 ± 2.04, female gonads 2.29 ± 0.75, liver 2.40 ± 0.35, and heart 1.65 ± 0.97 log CFU/g. During the course of the study, there was a slow decline in bacterial numbers with 3.00 ± 0.54 log CFU/g in spleen, 0.84 ± 0.58 log CFU/g in liver and 0.17 ± 0.43 log CFU/g in heart on day 28 pi, representing 97, 95, and 98% reductions, respectively. Although a decline in the number of bacteria was observed in head kidneys, this was not as pronounced as in the spleen, liver, and heart, and 2.69 ± 2.13 log CFU/g were present on day 28 pi (62% reduction). Following a decrease to 0.88 ± 1.10 log CFU/g on day 14 pi in the female gonads, an increase to 2.20 ± 1.77 log CFU/g was observed on day 28 pi. There was no correlation between spleen weight and CFU in spleen, and no difference in spleen weight or total body weight among infected and control fish (S3 Fig). Neither mortality nor macroscopic pathology was observed in infected fish. *Brucella pinnipedialis* HS strain was not found in either fecal or water samples.

### Infection with *B*. *pinnipedialis* HS strain induces expression of immune genes

**Fig 3:**
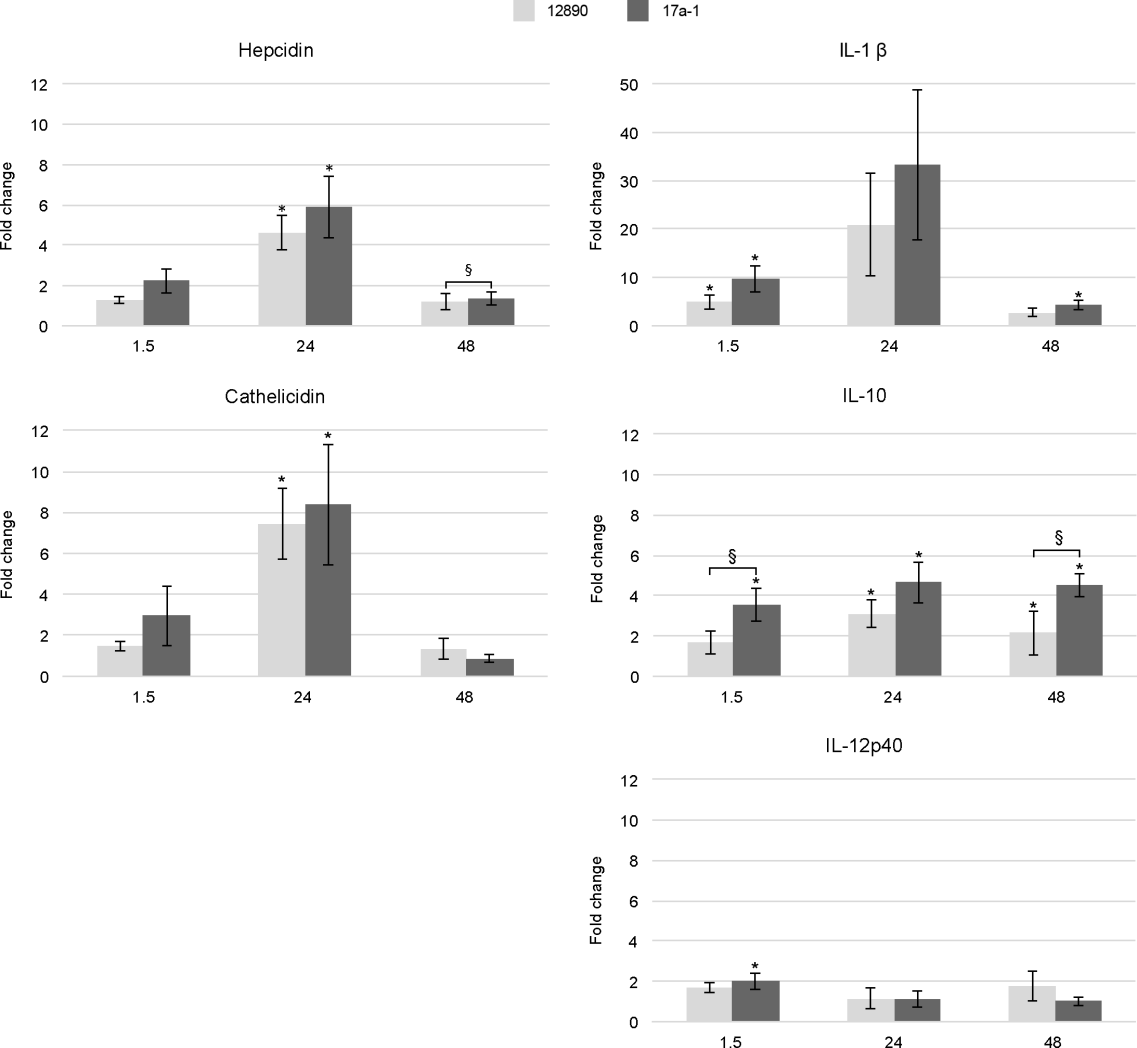
Expression of immune genes after *in vitro* infection with *Brucella pinnipedialis*. Relative gene expression of hepcidin, cathelicidin, interleukin (IL)-1β, IL-10, and IL-12p40 in cod head kidney derived monocytes/macrophages (HKDM) challenged with *B*. *pinnipedialis* reference (12890) and hooded seal (17a-1) strain at 1.5, 24 and 48 h post infection. The gene expression was normalized against the housekeeping gene 18S ribosomal RNA and calibrated against non-infected controls. Bars show the mean ± standard error of the mean of *n* = 4 fish. (*) Significantly different from non-infected controls, (§) 17a-1 significantly different from 12890 (*p* < 0.05 was considered significant).

RT-qPCR was used to measure expression of immune genes in cod HKDM challenged with *B*. *pinnipedialis* reference (12890) and HS (17a-1) strain, and in spleen from cod infected with *B*. *pinnipedialis* HS (17a-1) strain (S1 Table). Both *B*. *pinnipedialis* strains caused an increase in the expression of four of the five immune genes measured in HKDM cells. Upregulation of genes coding for the antibacterial peptides cathelicidin and hepcidin, and the cytokines IL-1β and IL-10 was most pronounced at 24 h pi (Fig 3). The expression of hepcidin, cathelicidin, and IL-10 was significantly increased compared to non-infected control cells treated otherwise similar. Due to large variations between individual fish, the expression of IL-1β was on the borderline of significance (*p* = 0.065 and 0.078, for *B*. *pinnipedialis* HS strain and *B*. *pinnipedialis* reference strain, respectively). Expression levels had returned to baseline by 48 h pi, with the exception of IL-10 that was still elevated. *Brucella pinnipedialis* HS strain generally induced greater expression of immune genes at all times pi than *B*. *pinnipedialis* reference strain in the cod HKDM. The exceptions were cathelicidin at 48 h pi and IL-12p40 where the levels were similar or lower. Although displaying a consistent trend, the only difference found significant was the expression of hepcidin at 48 h pi, and IL-10 at 1.5 and 48 h pi.

**Fig 4:**
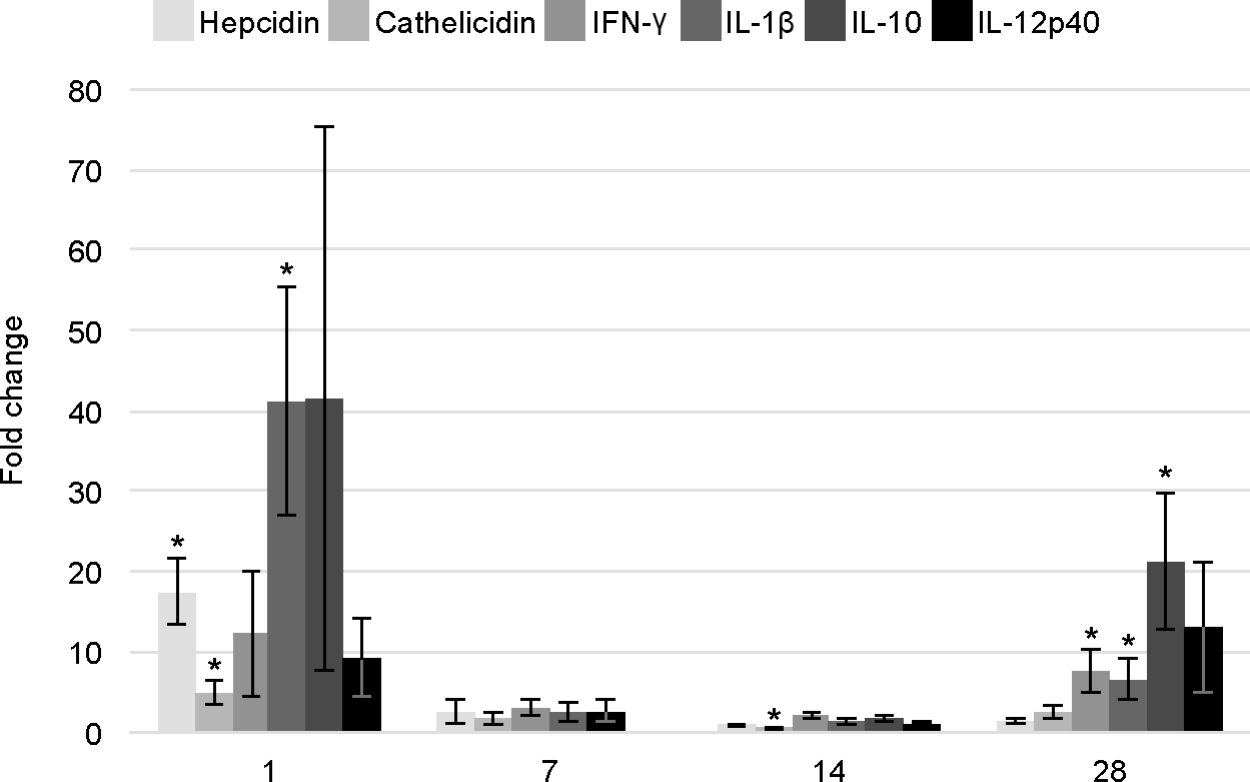
Expression of immune genes after *in vivo* infection with *Brucella pinnipedialis*. Relative gene expression of hepcidin, cathelicidin, interferon (IFN)-γ, interleukin (IL)-1β, IL-10, and IL-12p40 in spleen from cod infected with *B*. *pinnipedialis* hooded seal strain 17a-1 on days 1, 7, 14, and 28 post infection. The gene expression was normalized against the housekeeping gene 18S ribosomal RNA and calibrated against saline injected control cods. Bars show the mean ± standard error of the mean of *n* = 3–6 fish. (*) Significantly different from non-infected controls (*p* < 0.05 was considered significant).

Cod infected with *B*. *pinnipedialis* HS strain *in vivo* showed a general upregulation of cathelicidin, hepcidin, IFN-γ, IL-1β, IL-10 and IL-12p40 genes in spleen on day 1 pi compared to non-infected fish, but only hepcidin, cathelicidin, and IL-1β were significant (Fig 4). The expression of measured immune genes was only moderately induced at day 7 pi, with no significant changes. Expressions of the investigated immune genes had returned to baseline by day 14 pi, with the exception of cathelicidin, which was significantly downregulated at this point in time. On day 28 pi, a significant increase in the expression of IFN-γ, IL-1β, and IL-10 was again observed, with IL-10 being the most pronounced.

### Atlantic cod mounts a specific antibody response towards *Brucella*

**Fig 5:**
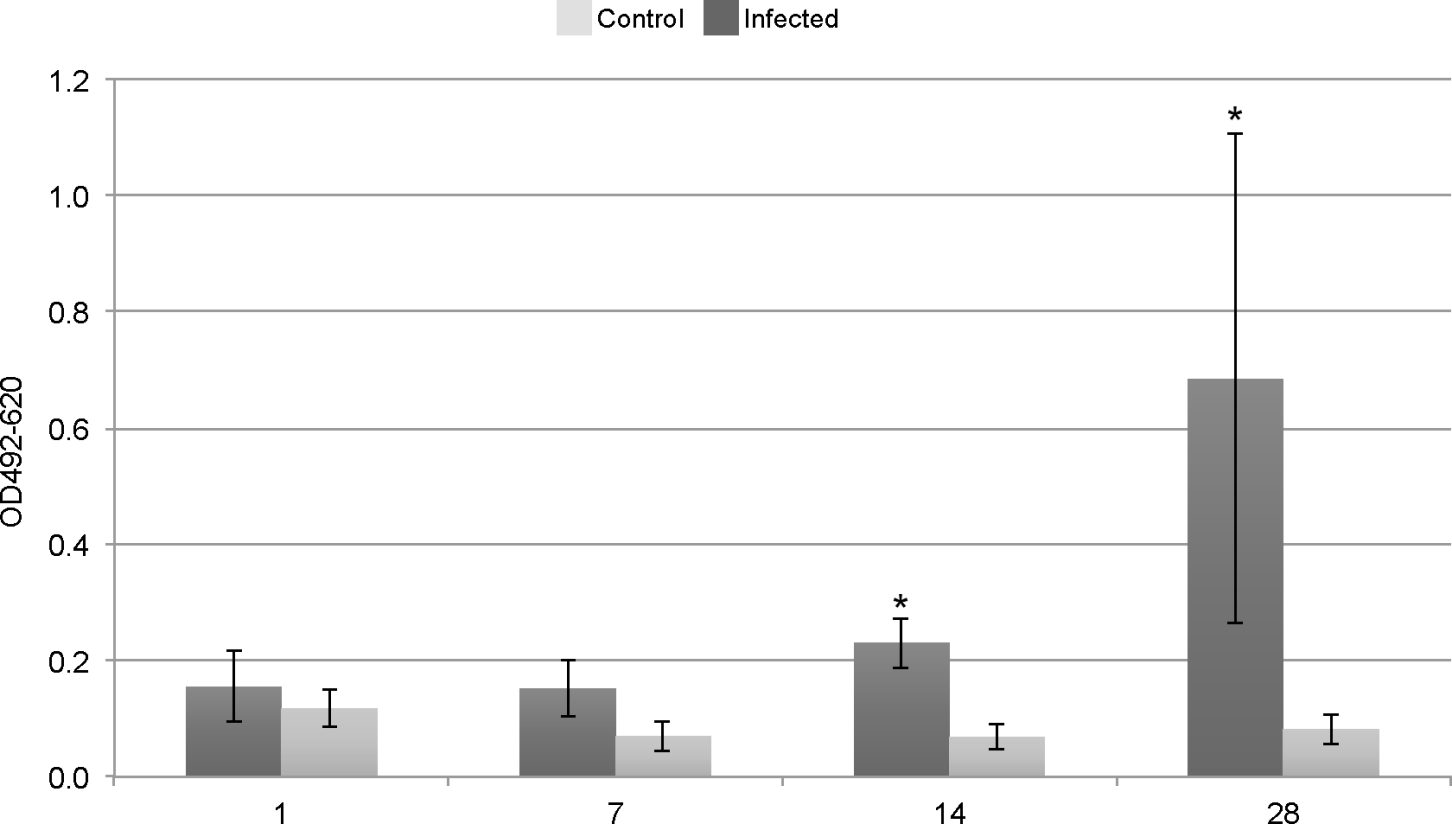
Atlantic cod infected with *Brucella pinnipedialis* mounts a specific antibody response. Level of anti-*Brucella* antibodies, as measured by an ELISA, in Atlantic cod after intraperitoneal injection of 7.5 x 10^7^ CFU of *B*. *pinnipedialis* hooded seal strain 17a-1 (dark grey) or sterile PBS (light grey) on days 1, 7, 14 and 28 post infection. Each bar shows the mean ± standard deviation of *n* = 4–6 fish. (*) Significantly different from non-infected controls (*p* < 0.05 was considered significant).

Amounts of specific anti-*Brucella* antibodies were determined using ELISA. The OD_620_ (mean ± SD) of all samples analyzed was 0.043 ± 0.004 indicating limited interference from nonspecific components. The OD_620_ of the sample dilution buffer control was 0.043 ± 0.002 and the OD_492_ was 0.057 ± 0.004, demonstrating a low background. The OD_492-620_ on days 1 and 7 pi for infected cod did not differ significantly from that of the control cod (Fig 5). On days 14 and 28 pi, however, the OD_492-620_ of infected cod (0.230 ± 0.044 and 0.684 ± 0.421) was significantly higher than that of controls (0.068 ± 0.022 and 0.082 ± 0.027).

## Discussion

Our study comprises the first experimental infection conducted in an Arctic marine fish species using a marine mammal strain of *Brucella*. By investigating whether *B*. *pinnipedialis* HS strain can establish an infection in Atlantic cod, we have addressed an element in the hypothesis of transmission of the bacteria via the food chain. Brucellosis in marine fish has not been described prior to this study and several scenarios following infection of cod with *B*. *pinnipedialis* HS strain can be proposed. First, *Brucella*-positive cod may be asymptomatic carriers without bacterial shedding to the environment. Second, infected cod may develop acute or chronic disease, possibly including transmission either horizontally or vertically, to other fish. Irrespective of the epizootiology of *B*. *pinnipedialis* in fish, for transmission to hooded seals via ingestion of cod, the bacteria need to persist in the fish for a while after initial colonization.

The experimental challenge showed that *B*. *pinnipedialis* HS strain did not induce disease in Atlantic cod. Gross pathology, including visible lesions like granulomas, was not observed, even though viable bacteria were recovered from nearly all examined tissues. This is in contrast to catfish infected with *B*. *melitensis* where cutaneous manifestations and moderate enlargement of liver and spleen were detected [16]. This may be due to differences in pathogenicity between classical *Brucella* species (e.g. *B*. *suis* 1330 and *B*. *melitensis* 16M) and marine mammal brucellae, as documented in the mouse model [9, 10].

The highest numbers of bacteria were found in the spleen and head kidneys. These are organs consisting mainly of leukocytes, including macrophages [26, 42, 43], and the result was hence not unexpected. The elimination of bacteria from most tissues sampled was slow, however, the protracted rate of elimination in the head kidneys compared to the spleen and liver was unpredictable. Substantial numbers of bacteria were still present in the head kidneys on day 28 pi. Additionally, bacteria survived intracellularly for an extended period of time in cod primary HKDM *in vitro*. This contrasts to observations made on primary alveolar macrophages from hooded seal where intracellular bacteria were eliminated within 48 h pi [13]. Whether this reflects differences in bactericidal mechanisms or other host-pathogen interactions in hooded seal and cod macrophages is currently unknown. Failure to completely eliminate bacteria can lead to an asymptomatic carrier state or chronic disease [44], and our results suggest that Atlantic cod is an asymptomatic carrier. For an infective disease as fish francisellosis, environmental conditions, in particular temperature, appear to play a significant role in the rate of morbidity and mortality [45, 46], however, it is presently not known to what extent increased environmental stress may affect the pathogenicity of a *B*. *pinnipedialis* infection in cod.

Together with impaired elimination from a host organism, multiplication of an infective agent will increase the chance of chronic infection. *Brucella pinnipedialis* HS strain is unable to multiply in hooded seal, murine or human macrophages, and human or hooded seal epithelial cells *in vitro* [12–14]. Additionally, HS strains 17a-1 and 22f-1 present a strongly attenuated pattern in the BALB/c mouse model (Jiménez de Bagüés and Nymo, unpublished data) and a previous study demonstrated a declining trend of CFU in spleen, liver and kidney with almost no bacteria present at six weeks pi in the same model [9]. In contrast to results from mammals where multiplication in later stages of the infection has not been documented, large numbers of bacteria were detected in female gonads in the later course of the infection in 2 out of 5 fish. Since these fish were not sampled prior to day 28, the elevated numbers could be due to a high colonization rate followed by a pronounced protracted bacterial elimination, and not multiplication. However, none of these two individuals displayed higher CFUs in other organs sampled compared to the rest of the group. This suggests that both impaired elimination and, possibly, multiplication of bacteria may occur in Atlantic cod. The presence of the bacteria in the female gonads also implies that horizontal transmission of *B*. *pinnipedialis* HS strain could be possible in Atlantic cod.

Detection of bacterial multiplication *in vitro* could be masked by release of intracellular bacteria into the gentamicin-containing media following cell death [47]; increased release of lactate dehydrogenase was detected at 48 h pi in the HKDM infection assay indicating cell damage. The latter was most likely due to a reduced capacity of primary HKDM to survive in culture [48], and not due to infection with *B*. *pinnipedialis*, as control wells were also affected to the same extent. Although not crucial in order to evaluate the potential of *B*. *pinnipedialis* to induce chronic infection, such an initial multiplication could have been detected *in vivo*, but head kidney tissue was not collected until day 7 pi.

The host’s immune response against the pathogen will contribute to how effective chronicity is established following invasion by the infective agent. *Brucella* spp. is described as a stealthy organism that has developed different strategies to avoid recognition by the mammalian immune system [49]. The intracellular lifestyle of *Brucella* limits exposure to the host innate and adaptive immune system. Several other factors including modification of pathogen-associated molecular patterns (PAMPs), reduced antigen presentation, and reduced activation of naïve T cells hamper an effective immune response and favor bacterial survival [50]. If *B*. *pinnipedialis* has characteristics that favor bacterial survival similar to pathogenic terrestrial brucellae, e.g. modified PAMPs and reduced antigen presentation, this could hamper the immune response following the invasion of host cells. In Atlantic cod, genes of several Toll-like receptors (TLR) that recognize bacterial surface antigens (TLR1, TLR2, TLR4, TLR5 and TLR6) are absent, whereas there may be increased functionality of major histocompatibility complex (MHC) I and other TLRs (TLR7, TLR8, TLR9, TLR22) [51]. With this in mind, a comparison of immune responses in mammals and fish would be speculative and possibly misleading, even more so in cod, a species in which MHC class II genes are absent [51], making comparisons between adaptive immune responses in mammals and cod virtually impossible. More research is necessary in order to understand how the cod immune system handles microbial pathogens, but this unique structure could contribute to the prolonged elimination of intracellular bacteria observed in Atlantic cod.

Immune cell activation in infected cod was proven by the observation of increased transcription of selected immune genes on day 1 pi, thereby demonstrating initiation of the innate immune response. Many antimicrobial strategies seen in mammals remain to be defined in bony fish, but multiple hepcidin isoforms responsible for iron deprivation can be found in various fishes [52]. Furthermore, hepcidin was transcriptionally upregulated in zebrafish (*Danio rerio*) on day 1 following infection with *Mycobacterium marinum* [53]. Cathelicidins in fish have a while ago been identified, but little is known about their function and importance in the immune system of fish. The gene expression of cathelicidin was upregulated on day 1 pi in Atlantic cod infected with *Aeromonas salmonicida* ssp. *achromogenes* [54], indicating a role in innate immunity. Infection with *B*. *pinnipedialis* HS strain in Atlantic cod resulted in significant upregulation of hepcidin and cathelicidin on day 1 pi, both *in vitro* and *in vivo*, demonstrating that iron deprivation and antimicrobial peptides play a role in the initial innate immune response against this marine *Brucella* sp.

The roles of IL-1β and IL-10 in regulating the inflammatory process are anticipated to be conserved in fishes [55]. Interestingly, the gene expression patterns, with a peak at 24 h pi, of IL-1β and IL-10 in cod HKDM after challenge with *B*. *pinnipedialis* HS strain were similar to what has been observed following challenges with *Francisella noatunensis* subsp. *noatunensis*, a known intracellular fish pathogen [27]. IL-1β and IL-10 were also upregulated in goldfish (*Carassius auratus)* kidney-derived monocyte/macrophage cultures *in vitro* and goldfish kidney tissue *in vivo* following infection with *M*. *marinum* [56, 57].

Type II IFN exerts regulatory roles in both innate and adaptive immunity. Teleost IFN-γ displays conserved functions compared to their mammalian orthologues and essentially contributes to the elimination of intracellular pathogens [58]. In contrast to the *in vitro* model, the gene expression patterns of IFN-γ, IL-1β and IL-10 in spleen of infected cod differed from those seen in cod infected with *F*. *noatunensis* subsp. *noatunensis* [59]. Cod infected with *B*. *pinnipedialis* HS strain displayed high expression on day 1 pi with a return to baseline on days 7 and 14 pi; significant increases in gene expression were seen in cod infected with *F*. *noatunensis* subsp. *noatunensis* after 7 and 14 days pi, but not on days 1–4 pi. A fast return to baseline also contrasts with observations in mice infected with *B*. *abortus* 2308, where both IL-12 and IFN-γ are increased for the first two weeks pi [60]. Several members of the IL-12/IL-23 subfamily are known in fishes and multiple paralogues of the different chains are present [58]. *Mycobacterium marinum* suppresses the production of IL-12p40 in human macrophages [61]. The expression of IL-12p40 was low in spleen tissue of cod infected with *B*. *pinnipedialis* HS strain on both 7 and 14 days pi, but downregulation was not observed.

Normalization of gene expression occurred before bacterial elimination from tissues was complete and observed CFUs in spleen were still high on days 7 and 14 pi. Normal immune gene expression was also observed in the HKDM cell model at 48 h pi, despite the presence of high bacterial CFUs. The lack of induced expression of immune genes in the spleen at these times could possibly be due to *Brucella* entering macrophages, hence hiding from other components of the immune system. Another possible explanation for the brief induction of immune genes could be a reduced pathogenicity of the *B*. *pinnipedialis* HS strain; the IFN-γ kinetic profile in mice depends on *Brucella* virulence and levels are shown to decrease faster after inoculation with attenuated *B*. *abortus* [60].

A significant increase in the gene expression of immune cytokines in spleen tissue was again detected on day 28 pi, indicating involvement of adaptive immune responses [62]. Both IFN-γ and IL-10 were significantly induced. The major adaptive immune response against intracellular bacteria is commonly anticipated to be cell-mediated immunity; however, the lack of MHC II, CD4, and invariant chain [51] most likely renders the canonical CD4^+^ pathway, including Th1, Th2, Treg and Th17 cells, absent from cod. Nevertheless, the p40 subunit of IL-12 might have a role in IL-12 promotion of proliferation and cytotoxicity of CD8^+^ cells [63, 64]. The increased expression of IL12p40 in spleen detected on day 28 pi may thus, combined with IFN-γ, lead to activation of cytotoxic T lymphocytes, an observation supported by the significant increase in IL-10 [65]. The changes in immune gene expression on day 28 pi were accompanied by a reduction in numbers of bacterial CFUs. Although the hiatus in immune gene expression in the spleen on days 7 and 14, until day 28 when expression resumes, is difficult to explain, it could be associated with a persistent *Brucella* infection. As stated by Grayfer and co-workers [52], “….*it is presently difficult to speculate whether changes in immune gene expression represent anti-bacterial host responses or if they reflect infection strategies of the intracellular pathogen*. *Further work is needed to decipher the respective host immune defence contributions and pathogen immune evasion strategies*.*”*

The immune system of Atlantic cod differs from that of several other bony fishes. Specific antibody responses were reported to be absent or low after immunization with *Vibrio salmonicida* [66] and *Vibrio anguillarum* [67]. Contrariwise, newer findings demonstrated specific antibody responses against inactivated *V*. *anguillarum*, *Aeromonas salmonicida*, as well as inactivated and live *F*. *noatunensis* subsp. *noatunensis* [41, 59], and now also against live *B*. *pinnipedialis*. Specific antibodies towards *Brucella* were detected on days 14 and 28 pi, confirming that the Atlantic cod mounts a specific humoral response towards the bacteria. Since Atlantic cod lacks the antigen presenting MHC II system [51], it is currently not known how humoral immune responses against bacterial infections are activated. In this study, antibodies were directed against epitopes associated with the O-polysaccharide chain of the smooth LPS of *Brucella*, as is the case in mammals infected with *Brucella* spp. [68, 69]. ELISA plates were coated with *B*. *abortus* LPS and *Brucella* LPS is known to be a T-independent antigen in mammals [70]. Thus the anti-LPS specific antibody response observed in cod is most likely caused by T-helper cell independent B-cell activation [71].

In addition to impaired elimination, in-host multiplication, and persistence promoting chronicity, shedding of bacteria from infected hosts with subsequent transmission to naïve hosts can contribute to the maintenance of a bacterial pathogen in a population [72]. *Brucella pinnipedialis* HS strain was not found in cod fecal matter and could not be detected in water collected from tanks with infected cod. Nonetheless, undetected bacterial shedding could still have taken place and direct transmission to in-contact fish cannot be completely ruled out.

In conclusion, our results show that *B*. *pinnipedialis* HS strain is capable of sustaining an asymptomatic infection in Atlantic cod for at least 28 days. Vertical transmission may take place, as there were indications of bacterial multiplication in, and/or pronounced protracted elimination from, female gonads. The lack of pathology associated with the persistent presence of *B*. *pinnipedialis* HS strain means that it should not be considered a pathogen for Atlantic cod in these conditions. However, our results indicate that the fish could act as a reservoir of *B*. *pinnipedialis* HS strain. Ingestion of Atlantic cod carrying *B*. *pinnipedialis* could, therefore, cause serologic conversion in hooded seals. To what extent marine mammal brucellae are present in wild fish has not been investigated. However, a scenario in which wild cod are carriers of *B*. *pinnipedialis* raises questions about whether environmental factors, such as increased water temperatures and persistent organic pollutants, could induce development of disease in infected fish and this may provide the basis for future research.

## Supporting Information

S1 Fig *Brucella pinnipedialis* survives in Atlantic cod head kidney derived monocytes/macrophages (HKDM). Intracellular survival of *B*. *pinnipedialis* hooded seal strain 17a-1 in cod HKDM at 1.5, 24, and 48 h pi. Cells were harvested from larger fish (1000 g) compared to the results in Fig 1. Results from each fish are depicted individually and each time point is the mean of 3 wells ± standard deviation. Cells harvested from fish number 2 did not meet the requirements with respect to density, morphology, and viability to be included in the infection assay.
(TIFF)

S2 Fig Atlantic cod head kidney derived monocytes/macrophages (HKDM) in culture release lactate dehydrogenase (LDH). The release of LDH increases with culture time irrespective of infection with *Brucella pinnipedialis* or not, without any difference between the reference (12890) and the hooded seal (17a-1) strain. The results are presented as percentage of total LDH (obtained by lysing cells in indicator wells at the same points in time as sampling). Each bar represents the mean of 2–3 wells ± standard deviation.
(TIF)

S3 Fig Infection with *Brucella pinnipedialis* HS strain does not affect spleen weight or growth rate. Weight of spleen (A) and total body (B) of control and infected fish given in gram (g) on days 1, 7, 14 and 28 post infection. No significant differences were found between control and infected fish. Each bar shows the mean ± standard deviation of *n* = 4–5 for control fish, and *n* = 5–6 for infected fish.
(TIFF)

S1 Table Expression of *immune* genes and bacterial counts after *in vitro* and *in vivo* infection with *Brucella pinnipedialis*. Ct values from real time PCR on cod head kidney derived monocytes/macrophages (HKDM; tab sheet named “*In vitro*”) from control wells and HKDM challenged with *B*. *pinnipedialis* reference (12890) and hooded seal (17a-1) strain. Number of colony forming units (CFU)/well are given. Ct values from real time PCR on spleen from saline injected control cod and cod infected with *B*. *pinnipedialis* hooded seal strain 17a-1 (tab sheet named “*In vivo*”). Number of CFU/ml blood and CFU/g organ are given.
(XLSX)
