# Supplementary figures and images for: Experimental Challenge of Atlantic Cod (Gadus morhua) with a Brucella pinnipedialis Strain from Hooded Seal (Cystophora cristata)
Source: PLoS One. 2016 Jul 14;11(7):e0159272. doi: 10.1371/journal.pone.0159272 (PMC4944957; doi:10.1371/journal.pone.0159272)

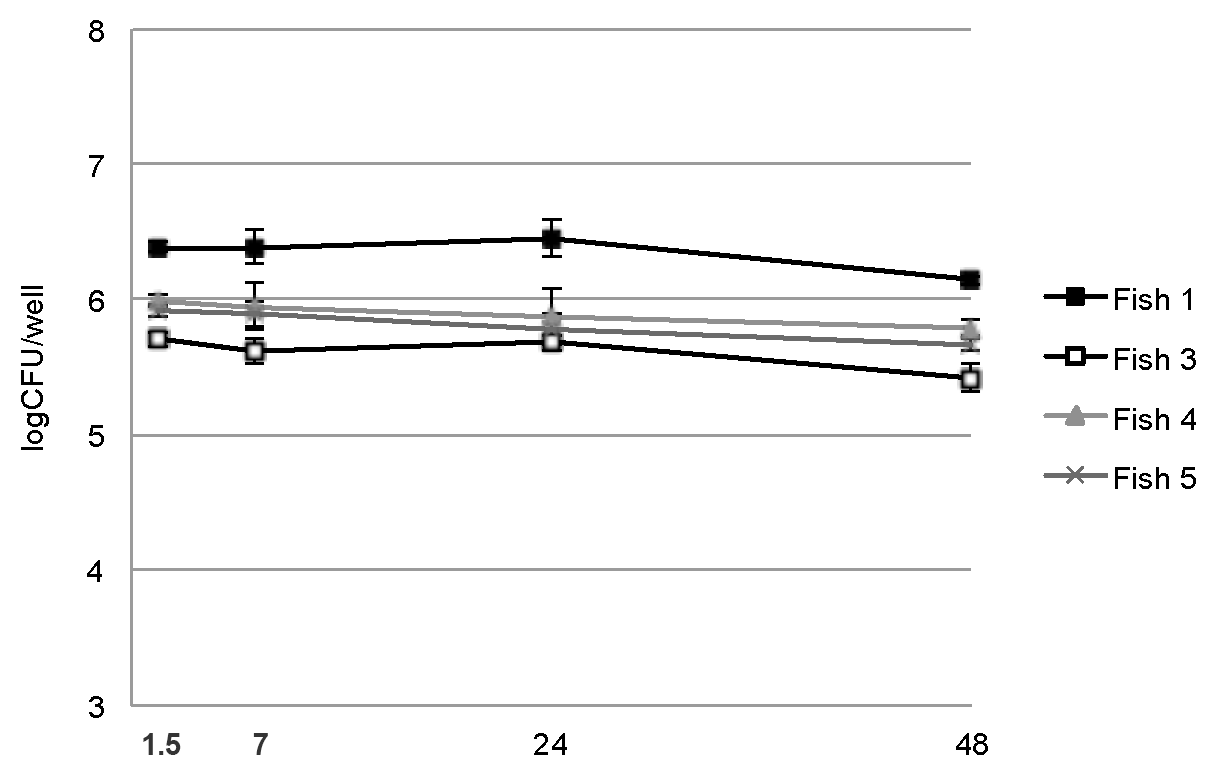

Supplement: S1 Fig — Intracellular survival of B. pinnipedialis hooded seal strain 17a-1 in cod HKDM at 1.5, 24, and 48 h pi. Cells were harvested from larger fish (1000 g) compared to the results in Fig 1. Results from each fish are depicted individually and each time point is the mean of 3 wells ± standard deviation. Cells harvested from fish number 2 did not meet the requirements with respect to density, morphology, and viability to be included in the infection assay. (TIFF) [file pone.0159272.s001.tiff]

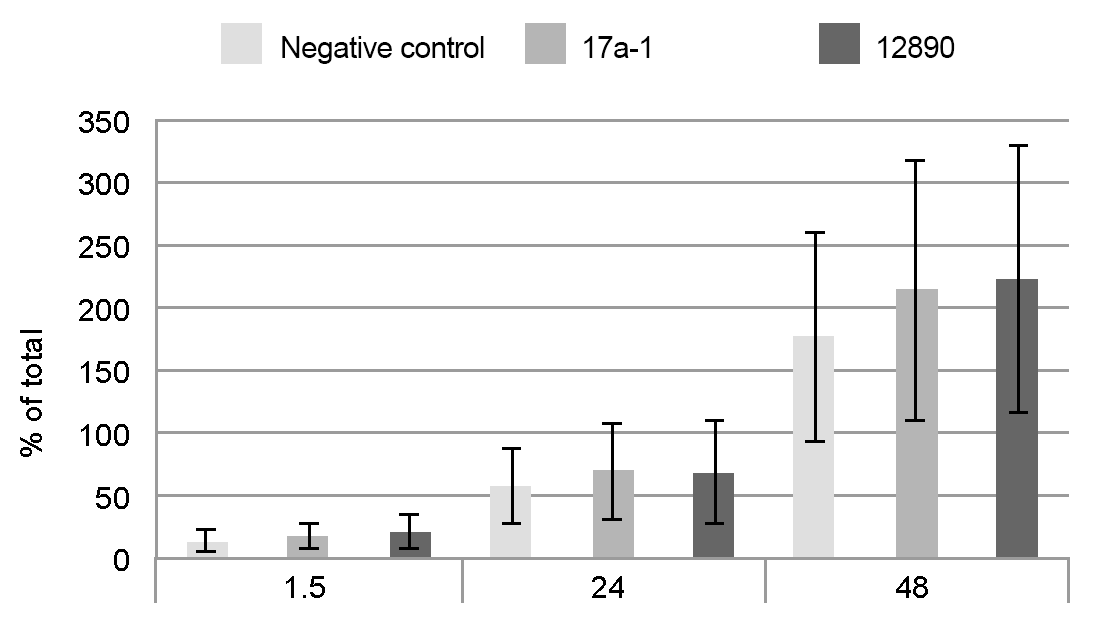

Supplement: S2 Fig — The release of LDH increases with culture time irrespective of infection with Brucella pinnipedialis or not, without any difference between the reference (12890) and the hooded seal (17a-1) strain. The results are presented as percentage of total LDH (obtained by lysing cells in indicator wells at the same points in time as sampling). Each bar represents the mean of 2–3 wells ± standard deviation. (TIF) [file pone.0159272.s002.tif]

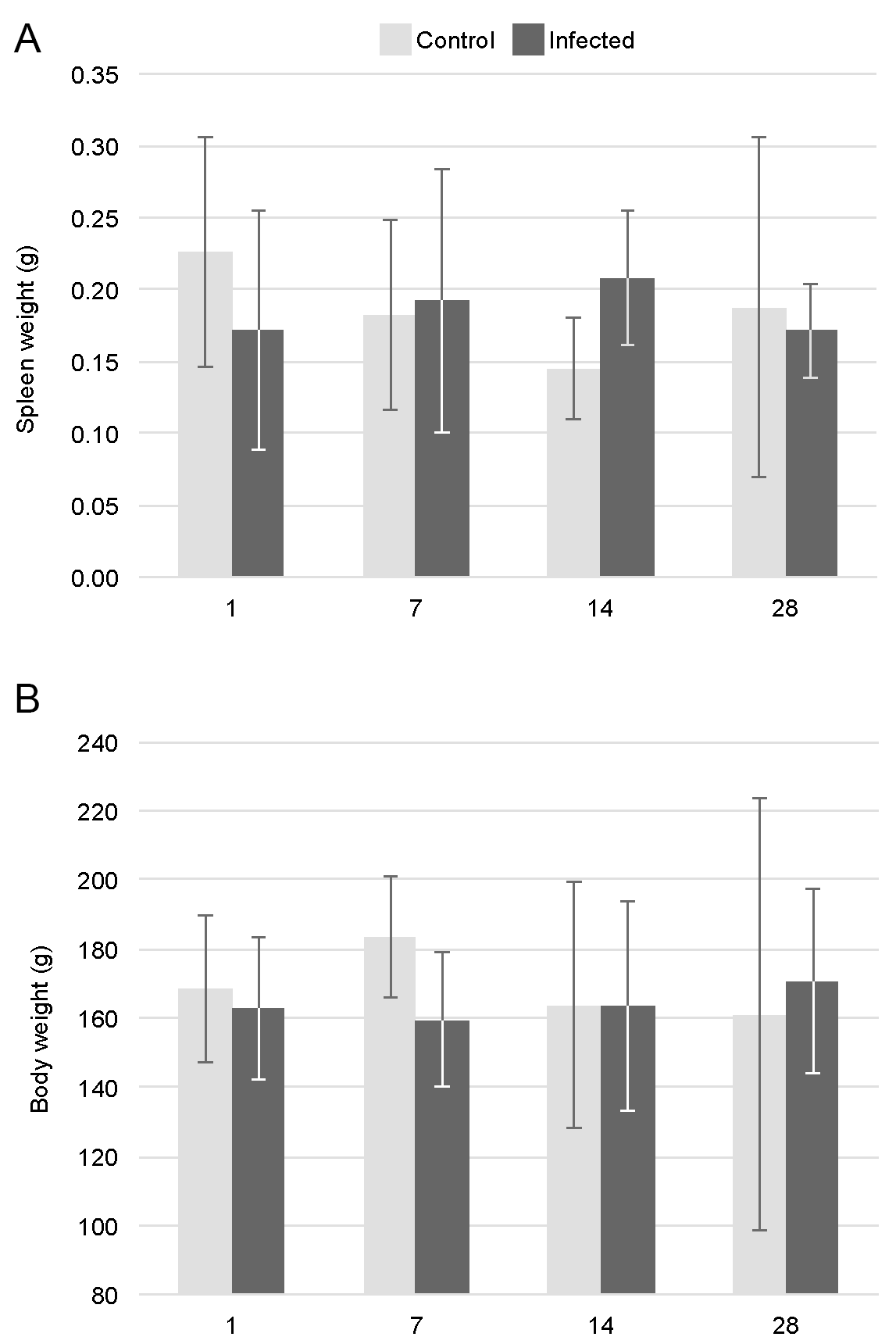

Supplement: S3 Fig — Weight of spleen (A) and total body (B) of control and infected fish given in gram (g) on days 1, 7, 14 and 28 post infection. No significant differences were found between control and infected fish. Each bar shows the mean ± standard deviation of n = 4–5 for control fish, and n = 5–6 for infected fish. (TIFF) [file pone.0159272.s003.tiff]
